# Supplementary material for: Identification and characterization of the WYL BrxR protein and its gene as separable regulatory elements of a BREX phage restriction system
Source: Nucleic Acids Res. 2022 May 2;50(9):5171–90. doi: 10.1093/nar/gkac311 (PMC9122589; doi:10.1093/nar/gkac311)
Supplement: gkac311_Supplemental_Files [file gkac311_supplemental_files.zip › SupplementaryFIguresCaptions.pdf]

SUPPLEMENTARY FIGURES

Supplementary Figure S1. *E. coli* ER2683 cells transformed with empty vector, vector encoding the WT BREX operon, or the same operon harboring precise deletions of each BREX protein factor. Reduced colony size and transformation efficiency is observed for ΔBrxL, ΔBrxB and ΔPglZ.

Supplementary Figure S2. Transcriptional start site (TSS) analysis of *Acinetobacter sp* NEB394 BREX operon. ***Panel a:*** Integrative Genomics Viewer (IGV) representation mapping PacBio SMRT-cappable-seq transcriptional start site data to the Acinetobacter BREX region in plasmid pBspH3. The *top track* shows position within the pBspH3 plasmid and the *second track* shows the BREX reading frames. The *third track* shows read coverage (gray, scale 0 to 3000) and *bottom track* shows mapped reads (scale 1 to 1000; note PacBio read strand output is arbitrary: reads in salmon map to forward strand, reads in blue map to reverse strand). This shows a strong TSS just before BrxR and the BREX operon, with only very low amounts of internal operon reads at various start positions, indicating one TSS for the entire operon. ***Panel b.*** SMRT-cappable-seq with a detailed view of the region upstream of the BrxR reading frame and the corresponding transcription start site. ***Panel c.*** Sequence of the Acinetobacter sp NEB394 BREX 5' UTR, indicating the location of the BrxR binding site (red, capitalized), predicted promoter region (underlined), transcription start site (+1, black arrow) and the start codon for the BrxR gene ('ATG' at +23).The same figure panel also used in **Figure 5b**.

Supplementary Figure S3. Phage restriction in *E. coli* NEB-5α by BREX. ***Panel a:*** Growth of *E. coli strain* NEB5α (New England Biolabs) transformed with pACYC184-BREX and challenged with λ_{vir} phage at MOI's ranging from 0.1 to 0.001. BREX$^-$ cells display lysis within five hours of the challenge, versus continued growth and saturation of the cells that harbor the BREX system. The level of protection conferred by the intact BREX system displayed a dependence on the phage MOI; cells containing BREX eventually crashed at the highest MOI (0.1) but continued to grow at MOI's of 0.01 and 0.001.

Supplementary Figure S4. *E. coli* strain ER2683 transformed with pACYC-BREX constructs containing the indicated single deletions were challenged with λ_{vir} phage at an MOI of 0.01. All BREX ORF's were required for restriction.

Supplementary Figure S5. Comparison of BrxR and PafBC structures. ***Panel a:*** PafBC comprises a tandem duplication of wHTH, WYL and WCX domains on the same polypeptide. The unbound form of PafBC (shown here) lacks the 2-fold symmetry present in the homodimeric structure of BrxR. Figure from Muller, 2019 (2). ***Panel b***: Overlay of PafBC's HTH-B domain with BrxR residues 1-71. ***Panel c:*** Overlay of PafBC's WYL-C domain with BrxR residues 119-193. ***Panel d:*** BrxR residues 211-291 aligned with PafBC's WYL-C domain; the domains are shown side-by-side for clarity. The domains share the same core topology (beta-beta-alpha-beta), indicated by numbering of secondary elements on the respective

domains. Regions A and B indicate elaborations on this core fold. In Region A (formed between beta(1) and beta(2)), BrxR contains an a-helix, whereas PafBC contains a b-strand and a-helix. In Region B (formed between helix(3) and beta(4), BrxR contains an a-helix, whereas PafBC contains a short linker.

Supplementary Figure S6. Electrophoretic mobility ('gel shift') analyses of BrxR interaction with 13 regions distributed across the BREX operon (positions indicated in top schematic). For each experiment (which are distributed across multiple gels in a composite figure below) lanes with a constant 10 nM concentration of dsDNA probe are incubated with 0 nM BrxR (left-most lane) and then a decreasing titration series of BrxR concentrations corresponding to 800, 400, 200 and 100 nM BrxR. In all cases, no observable specific binding is observed at 100 nM BrxR, and increased concentrations produce a non-specific smear of shifted DNA species.

Supplementary Figure S7. Electrophoretic mobility ('gel shift') analyses of BrxR interaction with various DNA probes spanning portions of the 5' untranslated region preceding the BREX operon. The relative truncations sites for the DNA probes used in the analysis are indicated below the schematic of the 5' UTR region. The analysis indicates the BrxR binding site (red uppercase font) corresponds approximately to basepairs -51 to -27 (relative to the BREX transcription start site; see **Supplementary Figure S2**). The -35 and -10 elements of a predicted bacterial promoter (the former of which overlaps with the BrxR binding site) are indicated by <u>underlined bases</u>. The BREX transcription start site at position +1 is indicated with the arrow; the BrxR translation start codon is indicated by the capitalized ATG.

Supplementary Figure S8. Overlay of the apo- (cyan) and DNA-bound forms of BrxR shows that the protein undergoes very minor conformational changes upon binding DNA. The two forms share an RMSD across all α-carbons of approximately 0.8 Å.

Supplementary Figure S9. Purification and characterization of BrxR point mutants. A series of six point mutations (R47A, which is located in the protein's DNA binding domain, and five additional constructs located in the WYL domain) were purified (***panel a***) and demonstrated to all elute at volumes corresponding to dimers on a size exclusion column (***panel b***). Subsequent determination of their thermal denaturation temperatures and unfolding behavior using CD spectroscopy demonstrated that one point mutant (R182A) was significantly destabilized (***panels c-d***). One point mutant in the WYL domain (R149A) was analyzed for DNA target binding and shown to interact with that site in a manner similar to the wild-type protein (***panel e***). The first three lanes in panel a are also shown in Figure 8a; in both cases they illustrate the outcome from the same experiment (purification of WT and R47A BrxR).

Supplementary Figure S10. WYL proteins upstream of defense islands in *V. cholera* are BrxR homologs. VchInd5, which was annotated by Legault et al as a "WYL" protein, shares ~25% amino acid

sequence identify with BrxR. The predicted Alpha-fold structure of VchInd5 (left) closely matches the experimentally determined structure of BrxR, demonstrating that the V. cholera WYL proteins are BrxR homologs.

1. Solovyev V, Salamov A. Automatic Annotation of Microbial Genomes and Metagenomic Sequences. In: Li RW, editor. Metagenomics and its Applications in Agriculture, Biomedicine and Environmental Studies. Happague NY: Nova Science; 2011. p. 61-78.

2. Muller AU, Leibundgut M, Ban N, Weber-Ban E. Structure and functional implications of WYL domain-containing bacterial DNA damage response regulator PafBC. Nat Commun. 2019;10(1):4653.

Figure S1

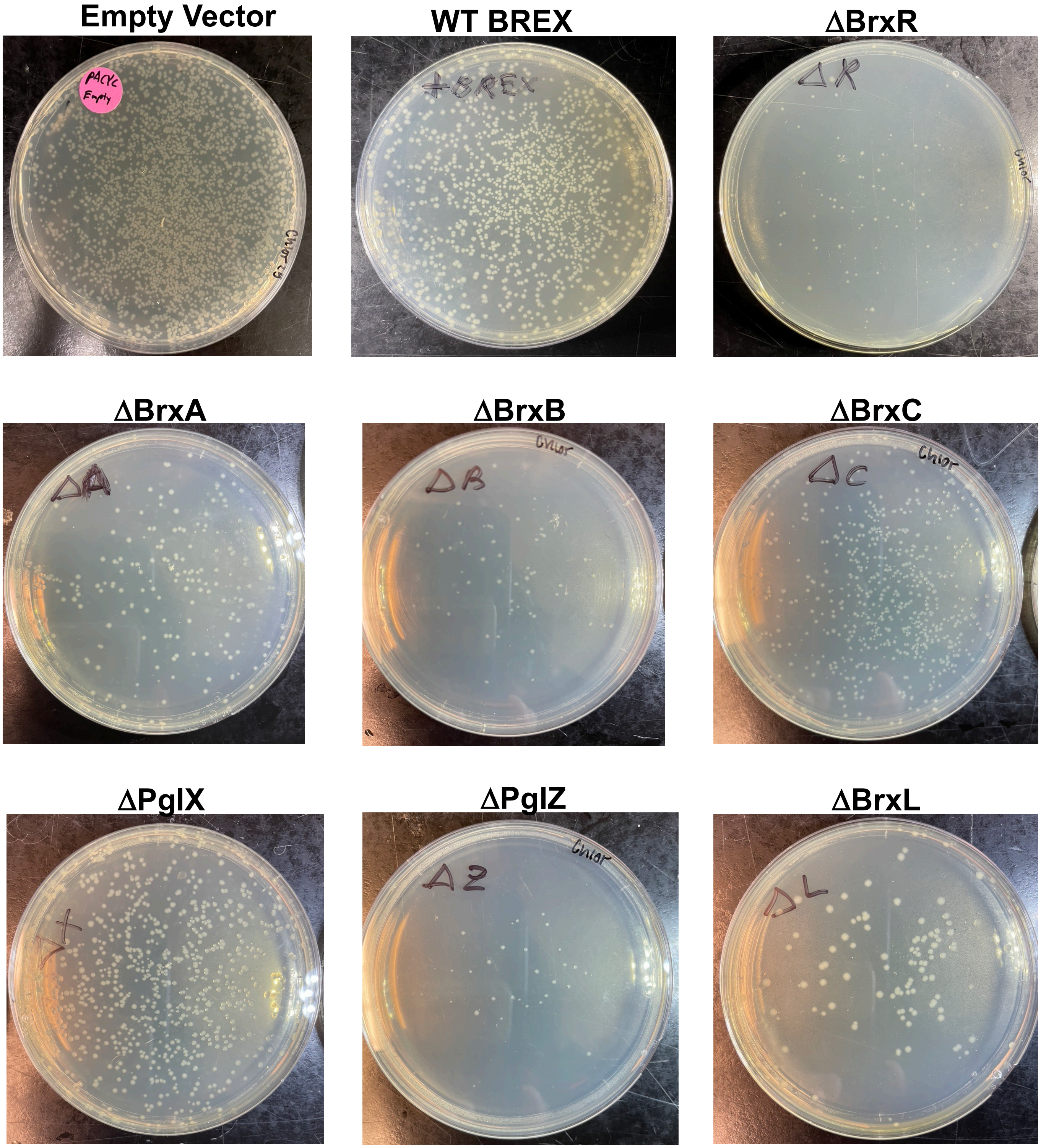
Empty Vector
WT BREX
ΔBrxR
ΔBrxA
ΔBrxB
ΔBrxC
ΔPglX
ΔPglZ
ΔBrxL

Figure S2

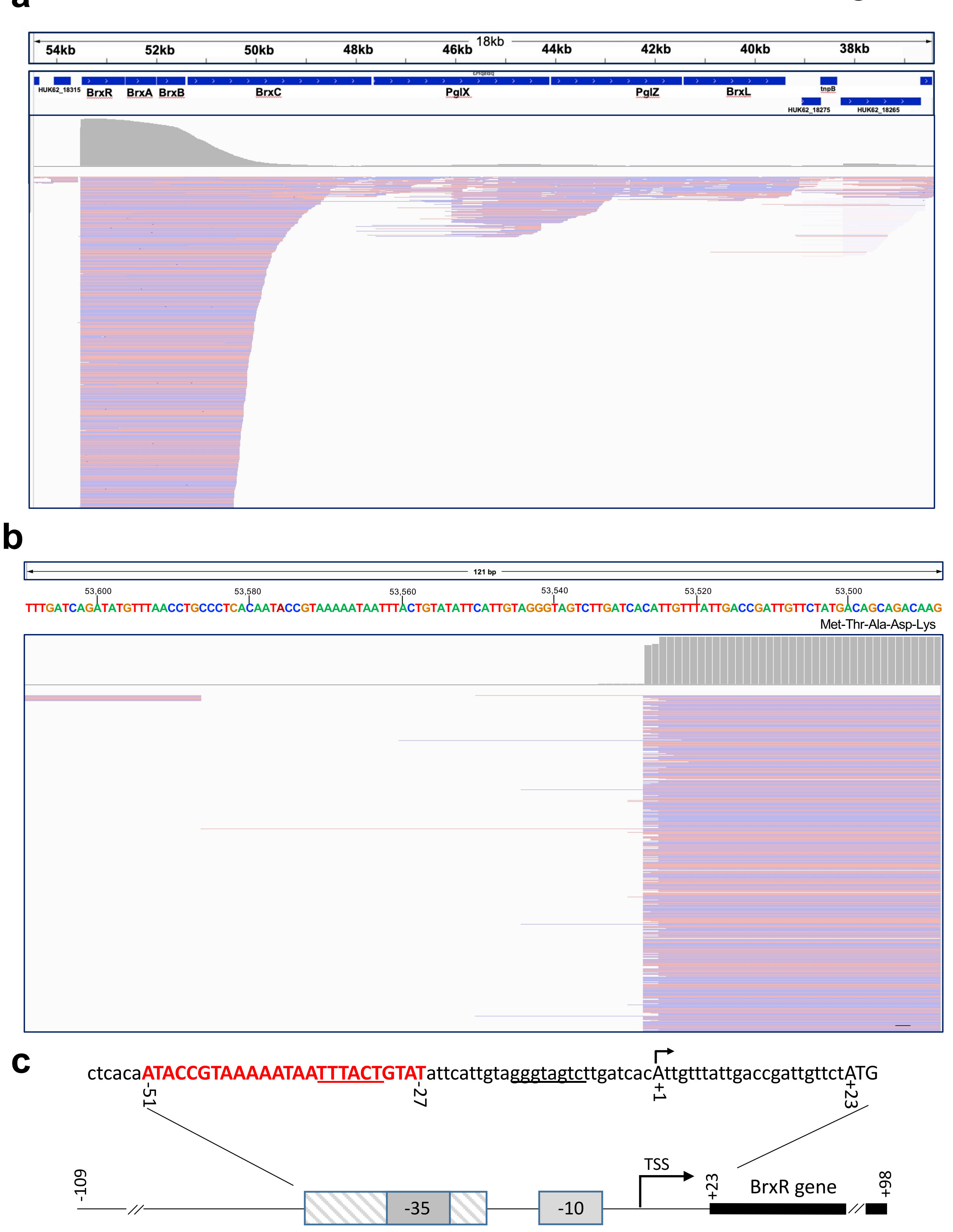
a
54kb
52kb
50kb
48kb
46kb
18kb
44kb
42kb
40kb
38kb
HUK62_18315
BrxR
BrxA
BrxB
BrxC
PglX
PglZ
BrxL
tnpB
HUK62_18275
HUK62_18265
b
121 bp
53,600
53,580
53,560
53,540
53,520
53,500
TTTGATCAGATATGTTTAACCTGCCCTCACAATACCGTAAAAATAATTTACTGTATATTCATTGTAGGGTAGTCTTGATCACATTGTTTATTGACCGATTGTTCTATGACAGCAGACAAG
Met-Thr-Ala-Asp-Lys
c
ctcacaATACCGTAAAAATAATTTACTGTATattcattgtagggtagtcttgatcacAttgtttattgaccgattgttctATG
-51
-27
+1
+23
-109
-35
-10
TSS
+23
BrxR gene
+98

Figure S3

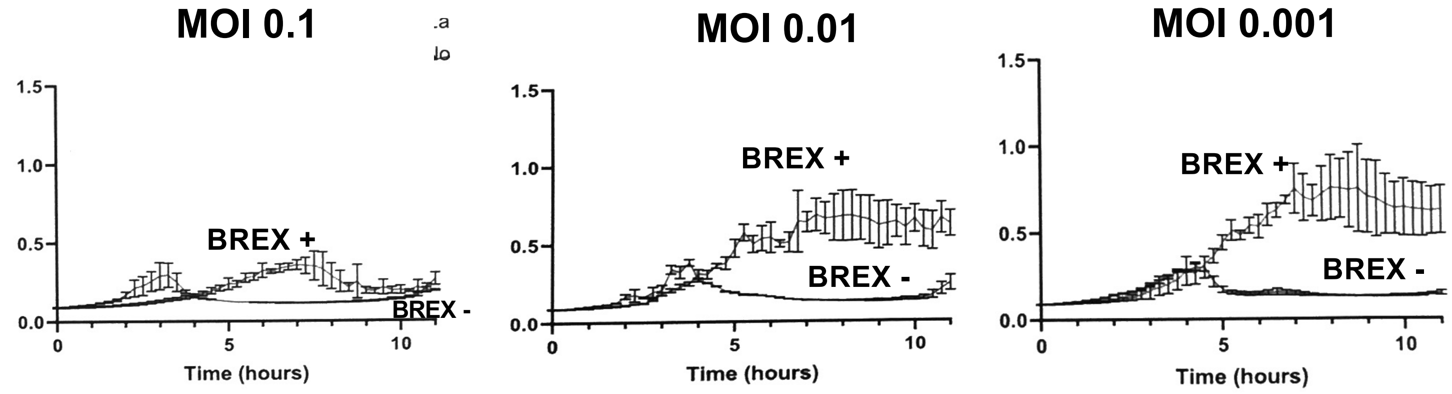

MOI 0.1
BREX +
BREX -
Time (hours)
MOI 0.01
BREX +
BREX -
Time (hours)
MOI 0.001
BREX +
BREX -
Time (hours)

Figure S4

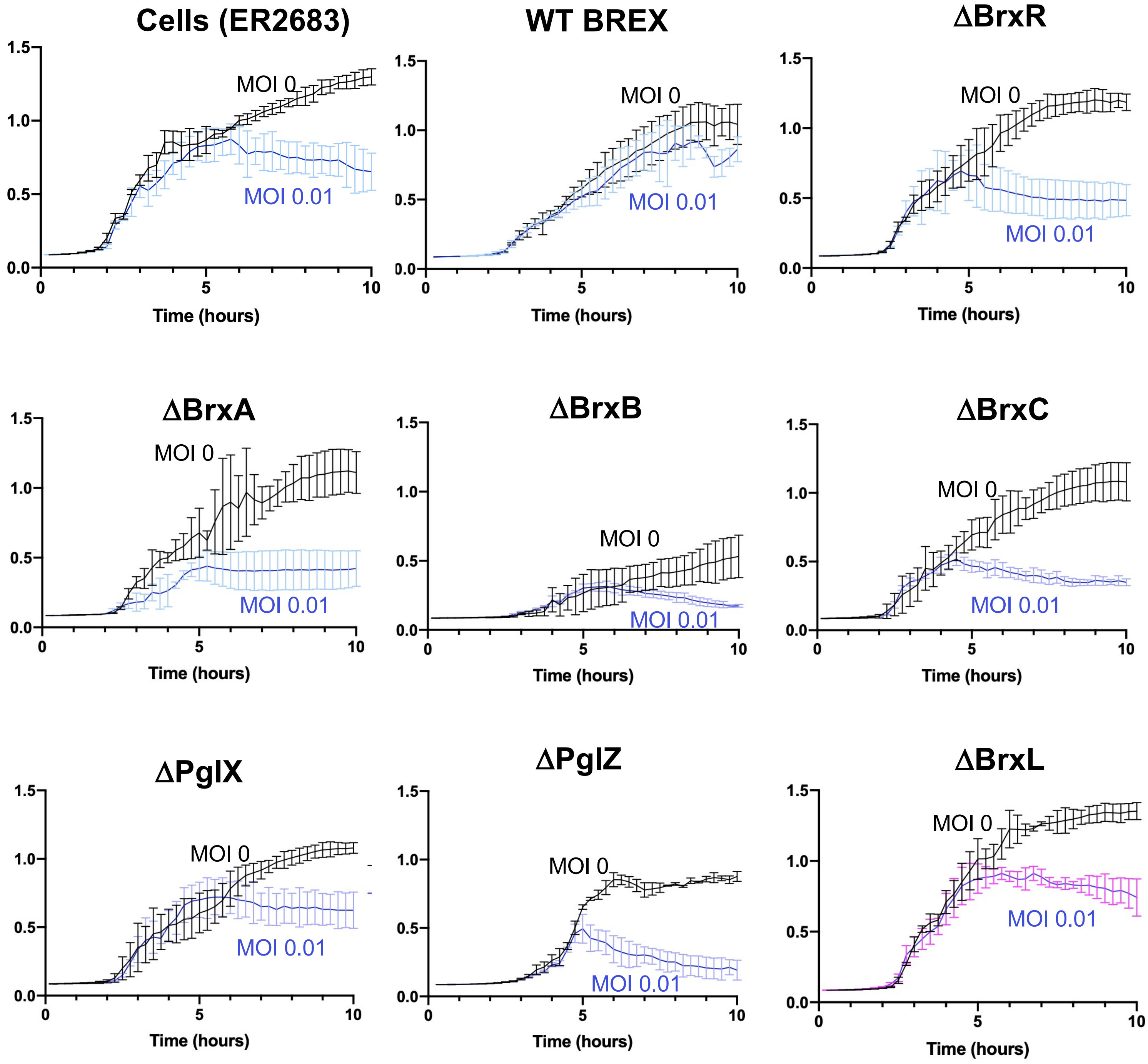
Cells (ER2683)
WT BREX
ΔBrxR
ΔBrxA
ΔBrxB
ΔBrxC
ΔPglX
ΔPglZ
ΔBrxL
MOI 0
MOI 0.01
Time (hours)
0.0
0.5
1.0
1.5
0
5
10

Figure S5

a

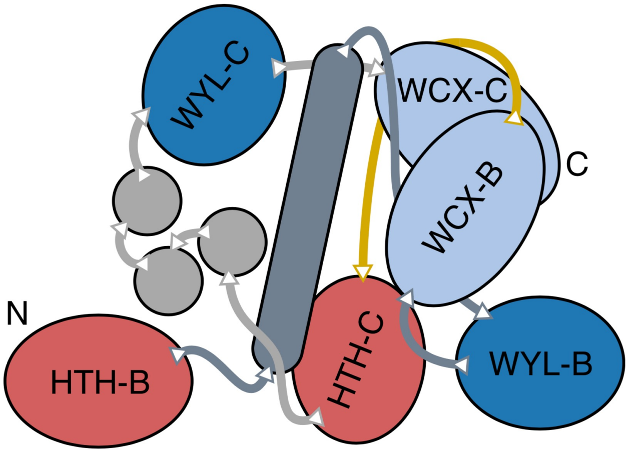

PafBC (Figure from Muller et al. Nature Comm. Vol. 10) (2019)

b **HTH**

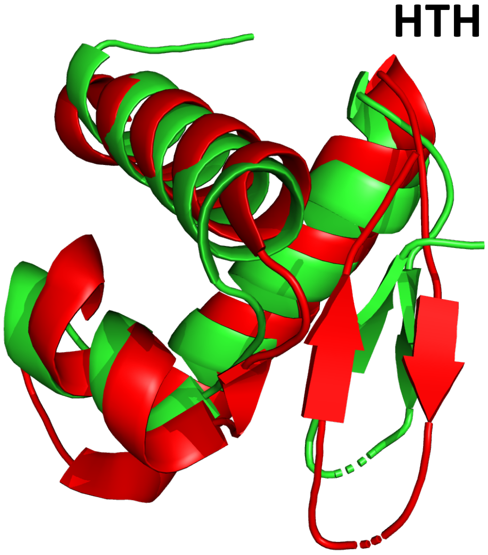

c **WYL**

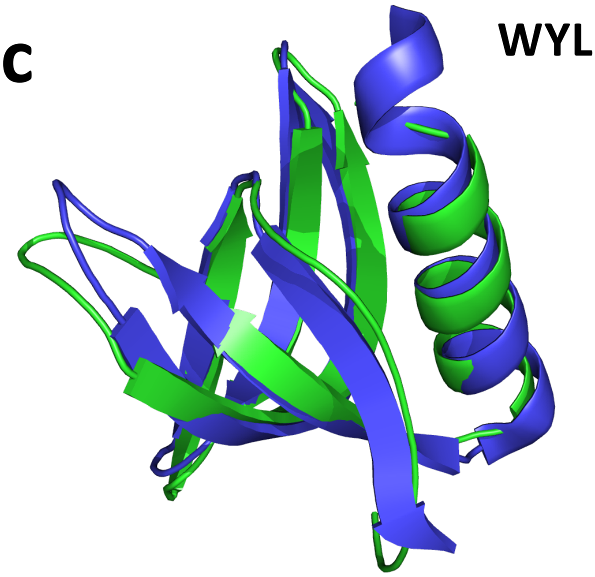

d

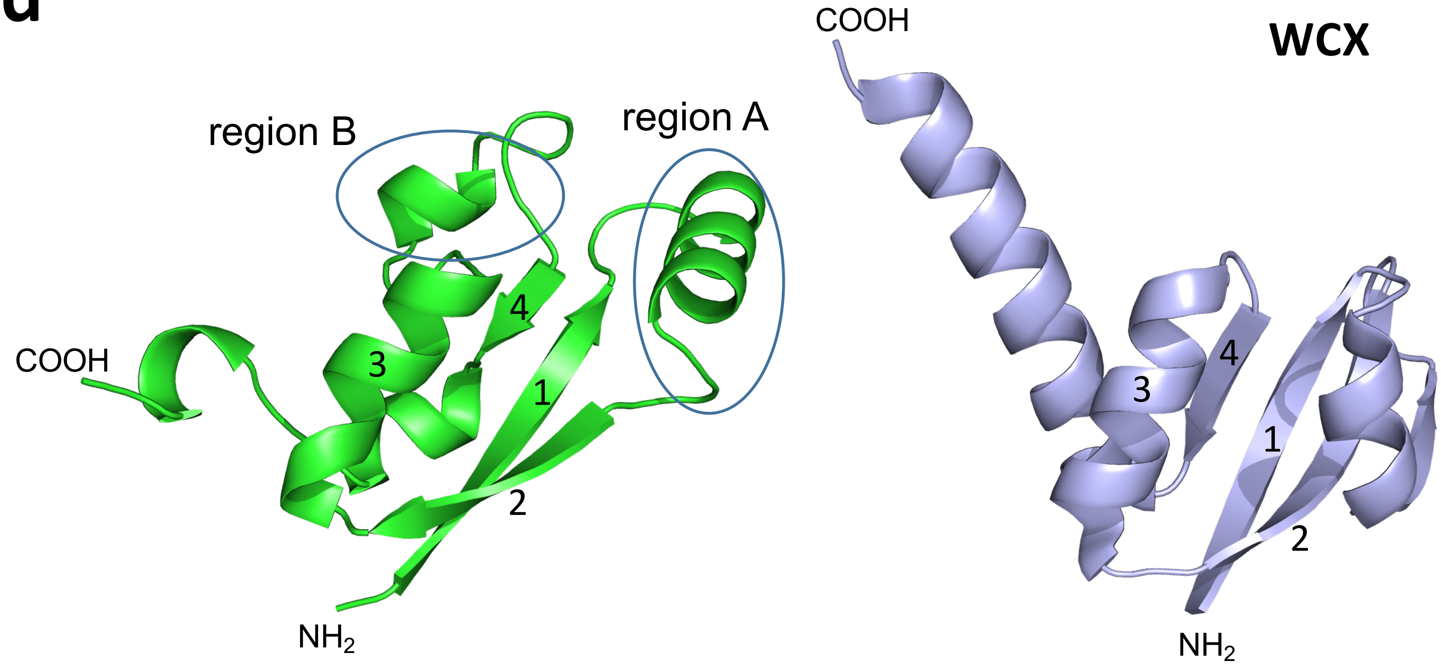

Figure S6

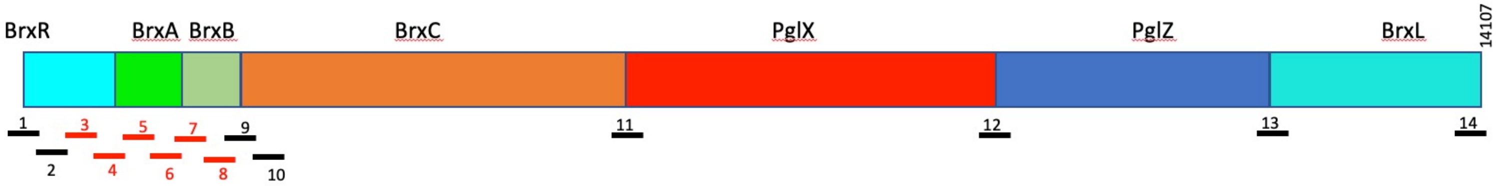

BrxR
BrxA
BrxB
BrxC
PglX
PglZ
BrxL
14107
1
2
3
4
5
6
7
8
9
10
11
12
13
14

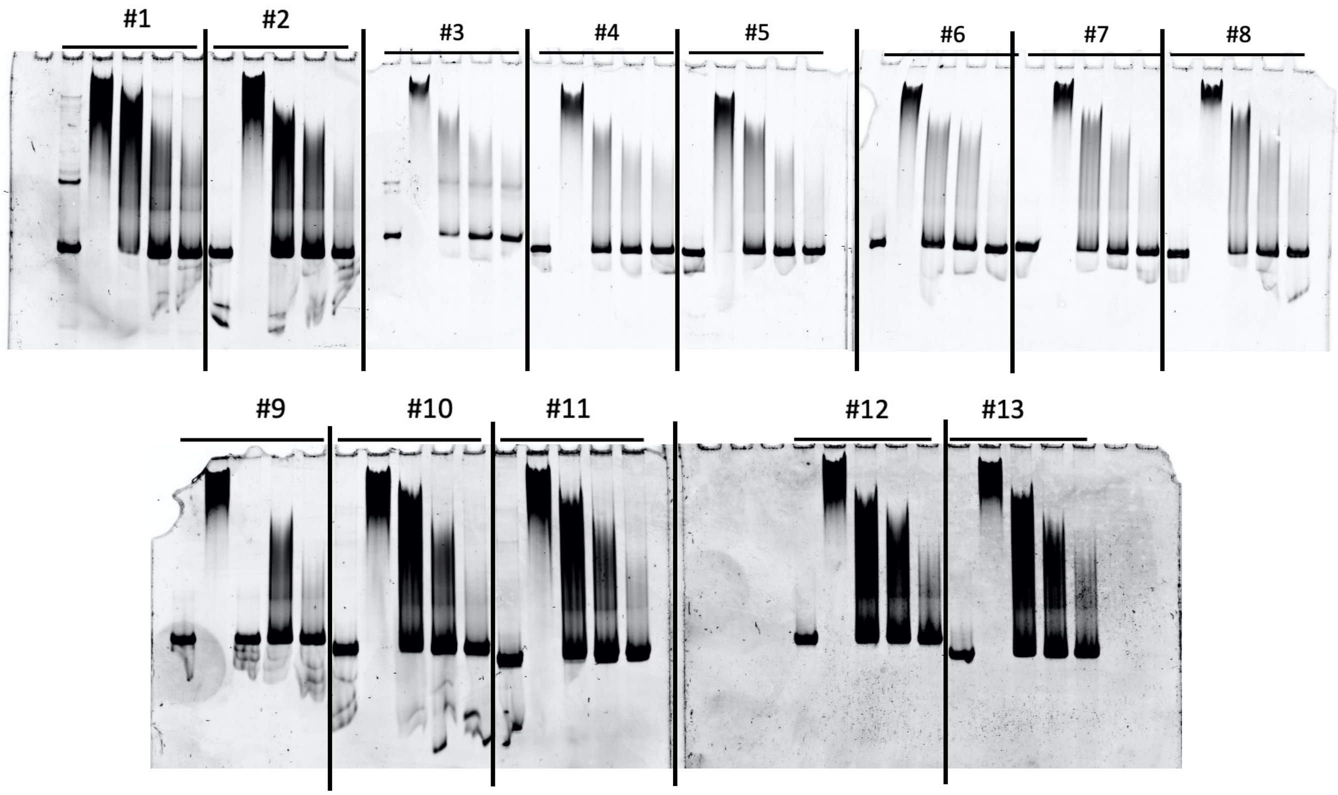

#1
#2
#3
#4
#5
#6
#7
#8
#9
#10
#11
#12
#13

Figure S7

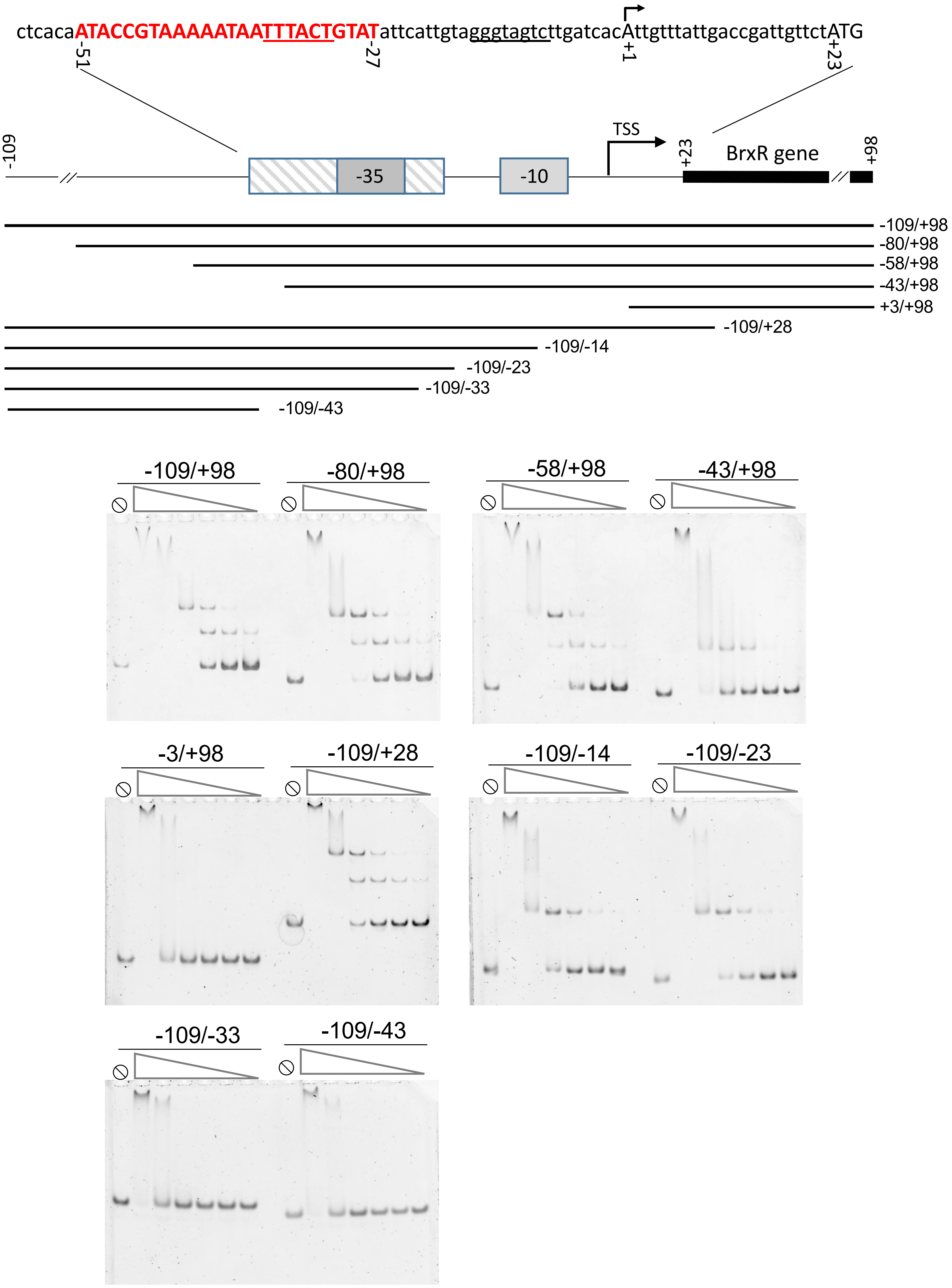

ctcacaATACCGTAAAAATAATTTACTGTATattcattgtagggtagtcttgatcacAttgtttattgaccgattgttctATG
-51
-27
+1
+23
-109
-35
-10
TSS
+23
BrxR gene
+98
-109/+98
-80/+98
-58/+98
-43/+98
+3/+98
-109/+28
-109/-14
-109/-23
-109/-33
-109/-43
-109/+98
-80/+98
-58/+98
-43/+98
-3/+98
-109/+28
-109/-14
-109/-23
-109/-33
-109/-43

Figure S8

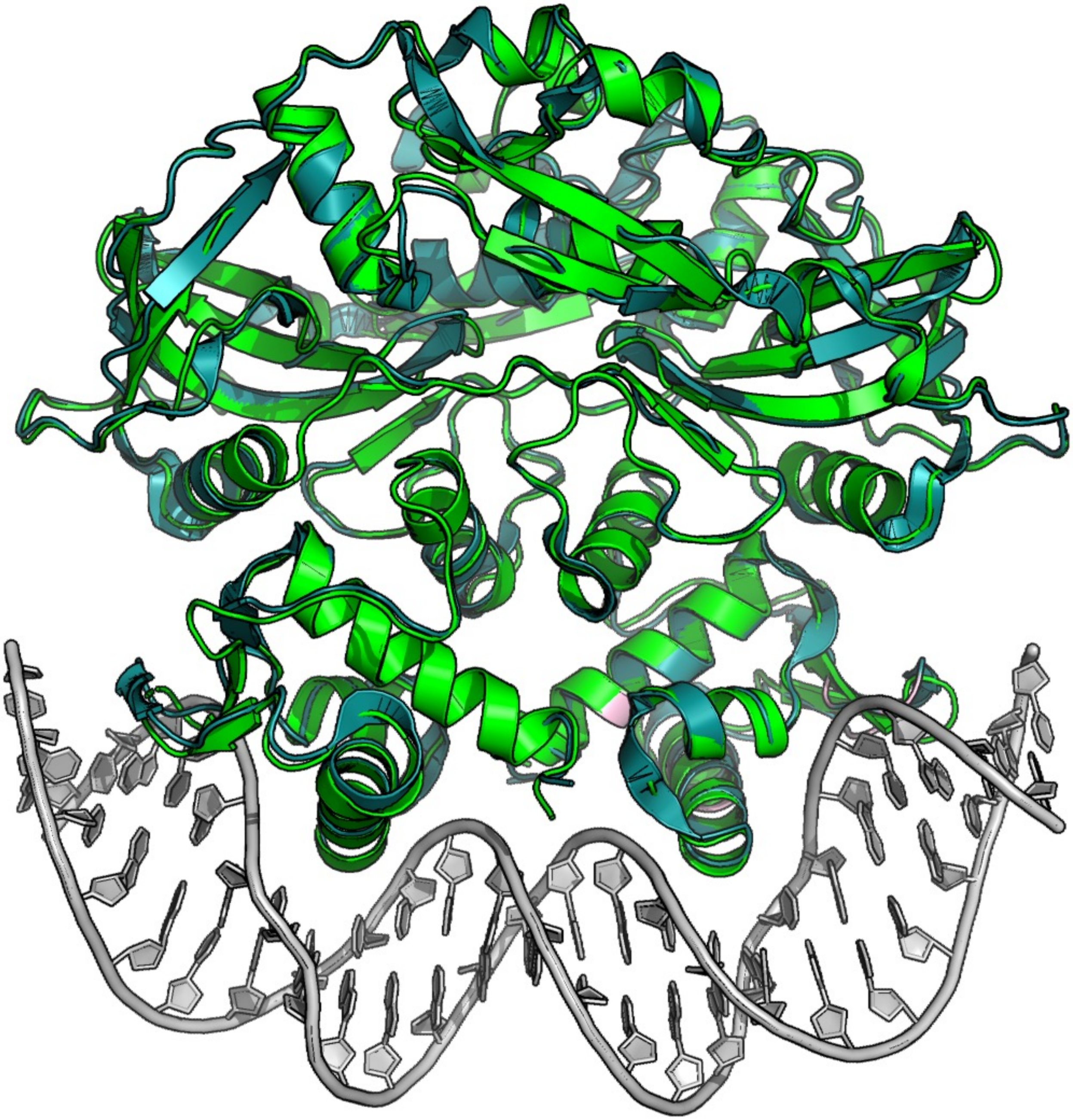

Figure S9

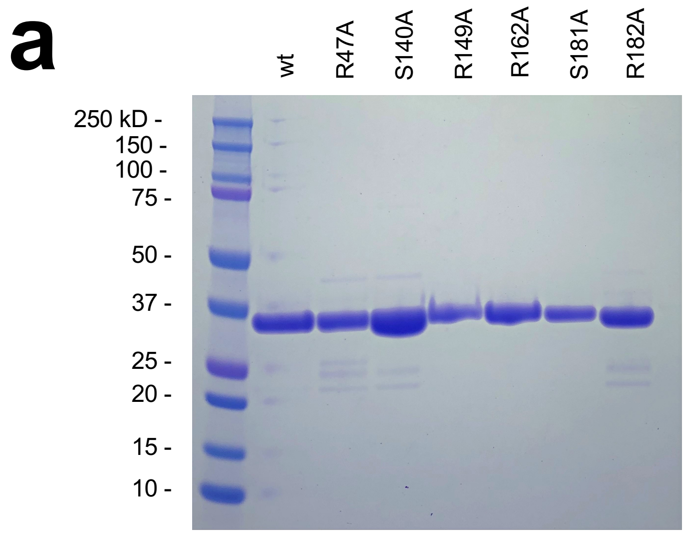

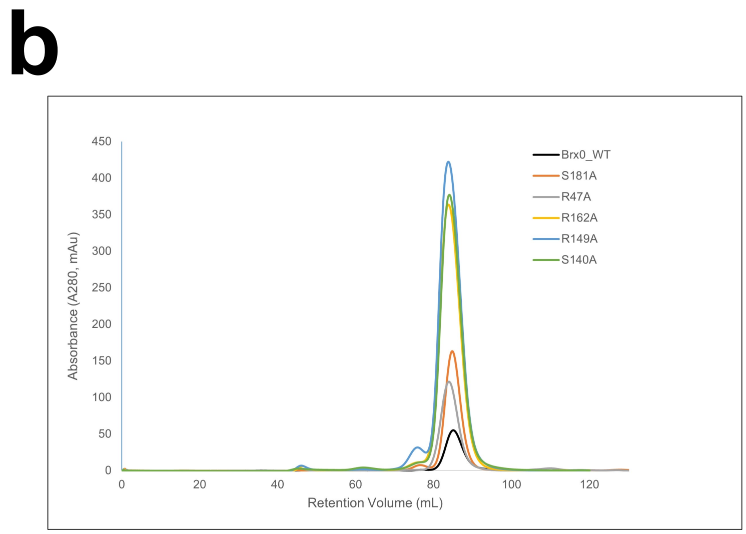

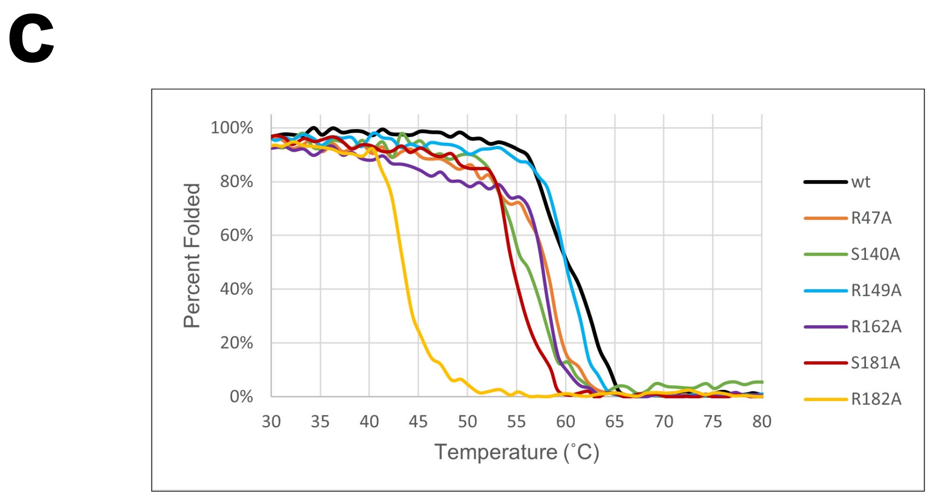

d

Construct	Tm (˚C)
Wild Type	60.3 +/- 0.054
R47A	58.1 +/- 0.073
S140A	58.7 +/- 0.080
R149A	60.9 +/- 0.051
R162A	58.0 +/- 0.800
S181A	55.1 +/- 0.050
R182A	43.7 +/- 0.053

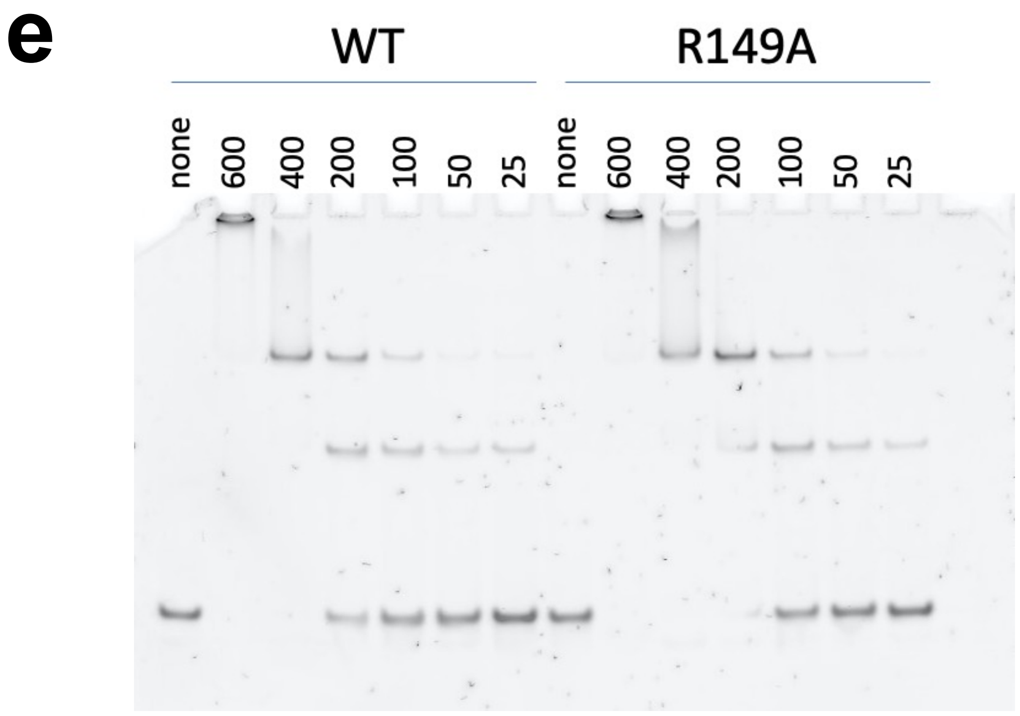

Figure S10

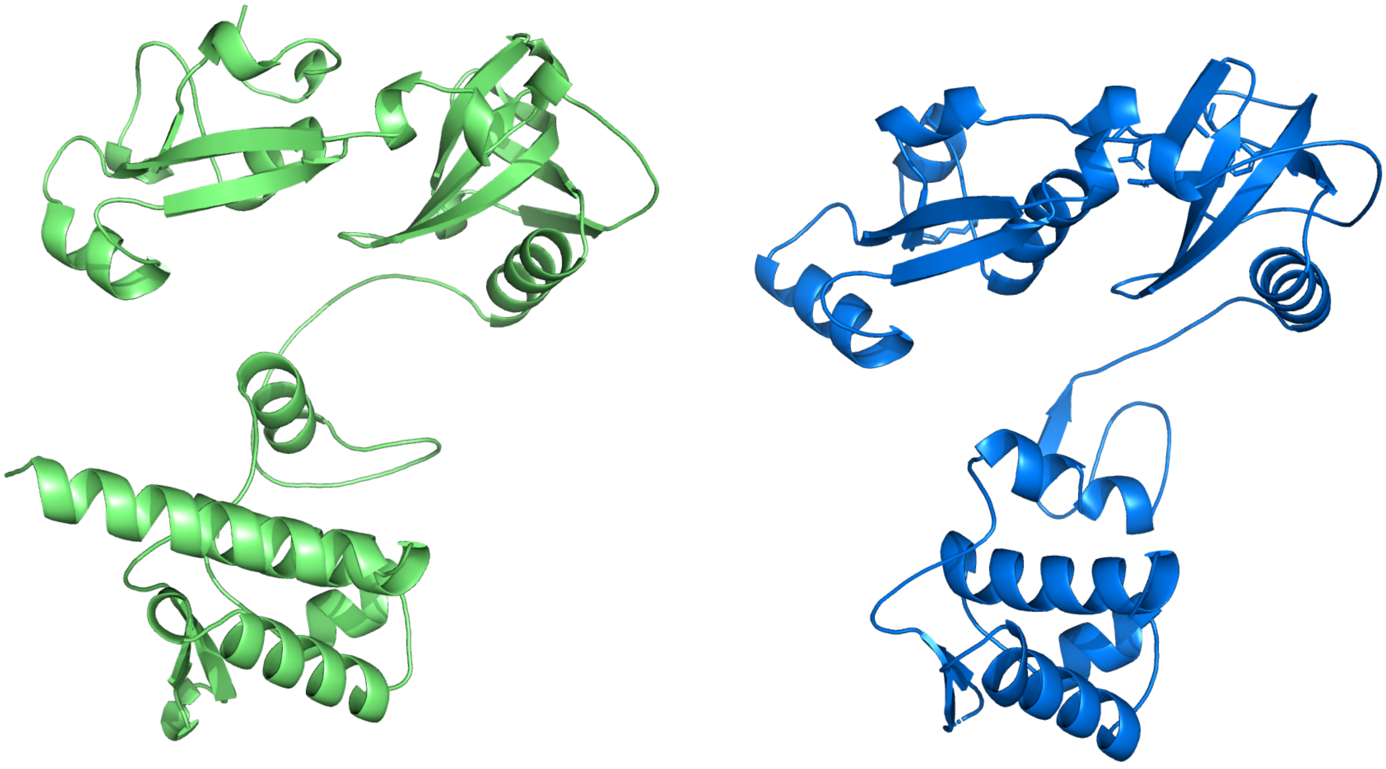

VchIND5 model (AlphaFold)

BrxR structure
