# Supplementary material for: Identification and characterization of the WYL BrxR protein and its gene as separable regulatory elements of a BREX phage restriction system
Source: Nucleic Acids Res. 2022 May 2;50(9):5171–90. doi: 10.1093/nar/gkac311 (PMC9122589; doi:10.1093/nar/gkac311)
Supplement: gkac311_Supplemental_Files [file gkac311_supplemental_files.zip › SupplementaryTableS1_Primers.pdf]

Supplementary Table S1. Primers for Molecular Biology and Subcloning and DNA constructs for binding assays

Primer Name	Oligonucleotide sequence (5’->3’)
**Ac_BREX block1 R**	5’-TCTAGATCTTCCCCGGGGATCCTTAAT CCACCCCTAACCCTTTAAACACC-3’
**Ac_BREX block1 F**	5’-ACTAGACCTTGATGAGGTCGTTGCATG-3’
**Ac_BREX block2 R**	5’-CATGCAACGACCTCATCAAGGTCTAGT-3’
**Ac_BREX block2 F**	5’-GACCAGCGACTGAGAAAGAGCTGTTTG-3’
**Ac_BREX block3 R**	5’-CAAACAGCTCTTTCTCAGTCGCTGGTC-3’
**Ac_BREX block3 F**	5’-TGCCTGCAGTTAAGGTTTAACATATGA CAGCAGACAAGCATGAAGTG-3’
**BrxA del R**	5’-ATTGCTGCCCCTCTACAAGGCCAATCT CCTATGAGGCCT-3’
**BrxA del F**	5’-TTGTAGAGGGGCAGCAATGAG-3’
**BrxB del R**	5’-TATGGCTATTTGTCGTGCTGCTGCCCC TCTACAACTAAC-3’
**BrxB del F**	5’-GCACGACAAATAGCCATAAAAATTCGG-3’
**BrxC del R**	5’-CGATATCCCTAACCCAAGCTCAATCAA TTCCCCGAATTTTTATGGCTA-3’
**BrxC del F**	5’-CTTGGGTTAGGGATATCGGCATC-3’
**PglX del R**	5’-GTACCGGAGAAAAAACATTCATTAAAAACCTTCAATTGATTGCTGATG-3’
**PglX del F**	5’-TGAATGTTTTTTCTCCGGTACTAG-3’
**PglZ del R**	5’-CGCTCTATCGTTAATTCGTTAGCAACG ACCTCATCAAGGTCT-3’
**PglZ del F**	5’-TAACGAATTAACGATAGAGCGTAGCCTTAC-3’
**BrxL del R**	5’-CTTCCCCGGGGATCCGTAGGCTACGCTCTATCGTTACGTTAT-3’
**BrxL del F**	5’-GGATCCCCGGGGAAGATCTAG-3’
**BrxR del R**	5’-TCTAACATGGCCAATCTCATGTTAAAC CTTAACTGCAGGCATGC-3’
**BrxR del F**	5’-GAGATTGGCCATGTTAGAACAATTTCATTA-3’
**R47A/g62a_LargeFrag1_forw**	5’-CACCGCCGGACATCAGCGCTA-3’
**R47A_LargeFrag1_rev**	5’-TGATATCTGCAGACGCCTGCTGACG-3’
**R47A_LargeFrag2_forw**	5’-CATGACCCCTCCGTCAAGGGTTATGT-3’
**R47A/g62a_LargeFrag2_rev**	5’-GCACGGACATGCCAGCGAAAAC-3’
**g62aSTOP_LargeFrag1_rev**	5’-GGCCAGTAACTCAATCGCCCTCATTC-3’
**g62aSTOP_LargeFrag2_forw**	5’-CTGGTTTGGTCTTAGCCGTCAGCAG-3’
**g62a_STOP_adaptor_top**	5’-GAATGAGGGCGATTGAGTTACTGGCCTACTAGGAAGGGCGTTTGGTCACCACAC-3’
**g62a_STOP_adaptor_bot**	5’-CTGCTGACGGCTAAGACCAAACCAGTTCATTAAACGTGTGGTGACCAAACGCCCTTC-3’
**R47A_Adaptor_top**	5’-CGTCAGCAGGCGTCTGCAGATATCAAACGCTACAATACGCTGTATAACCCAGATGCCTTG-3’
**R47A_Adaptor_bot**	5’-ACATAACCCTTGACGGAGGGGTCATGAATCAAGGCATCTGGGTTATACAGCGTATTGT-3’
**R47A/g62a_Secondary/Gibson_Forw**	5’-GGACATCAGCGCTAGCGGAGTGTA-3’
**R47A/g62a_Secondary/Gibson_Rev**	5’-GGACATGCCAGCGAAAACCGGTA-3’
**brxRBD_for1**	5’-**GGGTAGTCTTGATCACATTGTTTATTGACCGATTGTTCT**ATGACAGCAGACAAGCATGAA-3’
**brxRBD_delR_for1**	5’-**TGATCACATTGTTTATTGACCGATTGTTCT**ATGTTAGAACAATTTCATTATGACAGTGAC-3’
**brxRBD_rev1**	5’-**TACGGTATTGTGAGGGCAGGTTAAACATATC CAT**GAGAATTACAACTTATATCGTATGGG-3’
**brxRBD_for2**	5’-**CCGTAAAAATAATTTACTGTATATTCATTGTA**GGGTAGTCTTGATCACATTGTTTATTG-3’
**brxRBD_rev2**	5’-**TACAATGAATATACAGTAAATTATTTT**TACGGTATTGTGAGGGCAGG-3’
**brxRBD_delR_for2**	5’-**TTTACTGTATATTCATTGTAGGGTAGTCT**TGATCACATTGTTTATTGACCGATTG-3’
**brxRBD_delR_rev2**	5’-**ACCCTACAATGAATATACAGTAAATTATTTT**TACGGTATTGTGAGGGCAG-3’

Supplementary Table S1. Primers for Molecular Biology and Subcloning and DNA constructs for binding assays

To generate EMSA substrates to BrxR's upstream region (Figures 5, 8, S7)

**BrxR_ gblock_-109/+98, template**	GCGGAGTCCATCCAAACATCGGGAGATTTTGATCAGATATGTTTAACCTGCC CTCACAATACCGTAAAAATAATTTACTGTATATTCATTGTAGGGTAGTCTTGA TCACATTGTTTATTGACCGATTGTTCTATGACAGCAGACAAGCATGAAGTGC TTCTCAGAATGAGGGCGATTGAGTTACTGGCCTACTGGGAAGGGCGTTTG	Fig. 5, Fig 8. Fig S7
**BrxR_fwd_min109, primer**	CAAACGCCCTTCCCAGTAG	Fig. 5, Fig 8. Fig S7
**BrxR_rev_98, primer**	CAAACGCCCTTCCCAGTAG	Fig. 5, Fig 8. Fig S7
**BrxR_fwd_min, primer**	GATCAGATATGTTTAACCTGCCC	Fig. S7
**BrxR_fwd_min58, primer**	CTCACAATACCGTAAAAATAATTTAC	Fig. S7
**BrxR_fwd_min43, primer**	GCAGGAAATAATTTACTGTATATTCATTGTAGG	Fig. S7
**BrxR_fwd_20, primer**	GTTTATTGACCGATTGTTCTATGAC	Fig. S7
**BrxR_rev28, primer**	GTCATAGAACAATCGGTCAATAAAC	Fig. S7
**BrxR_rev28, primer**	GTCATAGAACAATCGGTCAATAAAC	Fig. S7
**BrxR_rev_min14, primer**	CCCTACAATGAATATACAGTA	Fig. S7
**BrxR_rev_min23, primer**	GAATATACAGTAAATTATTTTTACGG	Fig. S7
**BrxR_rev_min33, primer**	GTAAATTATTTTTACGGTATTGTGAG	Fig. S7
**BrxR_rev_43, primer**	TTTACGGTATTGTGAGGGC	Fig. S7

To generate off-target EMSA substrate (Fig. 5A)

**gblock_Off_target, template**	GTGCCAAAGTACTTTCGTTATAGCGGTGCAAGTACACCAATGCCTGGAAAG CACCTTGTTTACCACTGCTGAATAACCAGTAGATCGAGCGCTTTTTATAAGTC TGCATATGGTCTTTATAGAACTTGCTGGCTAAGTAACGGCGAATGGTGTCTT CAGCGGTTTCACTGGCTTTTTTATCCAAGCTTTCTGCCAGCCACAGCATATTG G	Fig. 5A
**Off_target_fwd_primer**	CCAATATGCTGTGGCTG	Fig. 5A
**Off_target_rev_primer**	GTGCCAAAGTACTTTCGTTATAG	Fig. 5A

To generate 25 bp BrxR EMSA substrates flanked by non-specific sequence (Fig. 6D).

**BrxR_target_panel1, template**	TTACGCTGGAGTCTGAGGCTCGTCCTGAATGATATaTACCGTAAAAATAATT TACTGTAtGATCTTACGGCATTATACGTATGATCGGTCCACGACAGC	Fig. 6D
**figure6d_panel_2, template**	TTACGCTGGAGTCTGAGGCTCGTCCTGAATGATATaTcCCGTAAAAATAATTT ACTGgAtGATCTTACGGCATTATACGTATGATCGGTCCACGACAGC	Fig. 6D
**figure6d_panel_3, template**	TTACGCTGGAGTCTGAGGCTCGTCCTGAATGATATaTACCcTAAAAATAATTT AgTGTAtGATCTTACGGCATTATACGTATGATCGGTCCACGACAGC	Fig. 6D
**figure6d_panel_4, template**	TTACGCTGGAGTCTGAGGCTCGTCCTGAATGATATaTACCGcAAAAATAATT TcCTGTAtGATCTTACGGCATTATACGTATGATCGGTCCACGACAGC	Fig. 6D
**fig6d_EMSA_fwd, primer**	TTACGCTGGAGTCTGAGGC	Fig. 6D
**fig6d_EMSA_rev, primer**	GCTGTCGTGGACCGATCATAC	Fig. 6D

To generate EMSA substrates to various regions in the BREX operon (Fig. S6)

**BrexAciOp1F_fig_S6, primer**	TGGACACGACTTGAGCTGCTAACGCATGCCTGC	Fig. S6
**BrexAciOp1R_fig_S6, primer**	TCAGCACAGCACTACGCCTGACAGCATATTAAGATATTCATTGATG	Fig. S6
**BrexAciOp2F_fig_S6, primer**	TGGACACGACTTGAGCAGCCTGTACTGACTACAGCG	Fig. S6
**BrexAciOp2R_fig_S6, primer**	TCAGCACAGCACTACGTGCTCTGATGGCAATAGGC	Fig. S6

Supplementary Table S1. Primers for Molecular Biology and Subcloning and DNA constructs for binding assays

**BrexAciOp3F_fig_S6, primer**	TGGACACGACTTGAGCGGTGTATACCGGTTTTCGCTG	Fig. S6
**BrexAciOp3R_fig_S6, primer**	TCAGCACAGCACTACGATTGCGGTCTGATATCGCTG	Fig. S6
**BrexAciOp4F_fig_S6, primer**	TGGACACGACTTGAGCTGAAAAAAGCCCTGGCTCAC	Fig. S6
**BrexAciOp4R_fig_S6, primer**	TCAGCACAGCACTACGGAATATTGTCAATCTGAATTGCCTGATG	Fig. S6
**BrexAciOp5F_fig_S6, primer**	TGGACACGACTTGAGCGAAGCGACTCCAGAACGATG	Fig. S6
**BrexAciOp5R_fig_S6, primer**	TCAGCACAGCACTACGTTCATCCAAAAAATCTGACCAGATATAAC	Fig. S6
**BrexAciOp6F_fig_S6, primer**	TGGACACGACTTGAGCGGCGCAATACTTGGACAGTTATATC	Fig. S6
**BrexAciOp6R_fig_S6, primer**	TCAGCACAGCACTACGTATTGTTTGACTCATTGCTGCCC	Fig. S6
**BrexAciOp7F_fig_S6, primer**	TGGACACGACTTGAGCGTCGGTATGGACGC	Fig. S6
**BrexAciOp7R_fig_S6, primer**	TCAGCACAGCACTACGTCGCTTTATCAATGAAATTGCG	Fig. S6
**BrexAciOp8F_fig_S6, primer**	TGGACACGACTTGAGCCAAGCAGTGGTGGATTACCTG	Fig. S6
**BrexAciOp8R_fig_S6, primer**	TCAGCACAGCACTACGTGGCCTGACCGTCGTAATAC	Fig. S6
**BrexAciOp9F_fig_S6, primer**	TGGACACGACTTGAGCGCATAAACCAGTCGTATTGTTTTATCC	Fig. S6
**BrexAciOp9R_fig_S6, primer**	TCAGCACAGCACTACGAAAGTCACGAAAATGCTTTTCCAG	Fig. S6
**BrexAciOp10F_fig_S6, primer**	TGGACACGACTTGAGCATGAGTACGTGGTCACCAATG	Fig. S6
**BrexAciOp10R_fig_S6, primer**	TCAGCACAGCACTACGATGACATCGGCATGATGACTAAC	Fig. S6
**BrexAciOp11F_fig_S6, primer**	TGGACACGACTTGAGCCAGCAAAAGCTCAAGTAGTGTCTATC	Fig. S6
**BrexAciOp11R_fig_S6, primer**	TCAGCACAGCACTACGGCAAGTCGCCTTTCTGTTCAG	Fig. S6
**BrexAciOp12F_fig_S6, primer**	TGGACACGACTTGAGCCACTATGCCGATCAGCGTATTAG	Fig. S6
**BrexAciOp12R_fig_S6, primer**	TCAGCACAGCACTACGGCCATATTTAAAATATGGACATCATTTAGC	Fig. S6
**BrexAciOp13F_fig_S6, primer**	TGGACACGACTTGAGCCAGGTCAGACTGTCATTGTCTG	Fig. S6
**BrexAciOp13R_fig_S6, primer**	TCAGCACAGCACTACGGGTATTCGAGTACATAGACCGGG	Fig. S6
